# Cost-effectiveness of molecular point-of-care testing for influenza viruses in elderly patients at ambulatory care setting

**DOI:** 10.1371/journal.pone.0182091

**Published:** 2017-07-27

**Authors:** Joyce H. S. You, Lok-pui Tam, Nelson L. S. Lee

**Affiliations:** 1 School of Pharmacy, Faculty of Medicine, The Chinese University of Hong Kong, Shatin, Hong Kong, China; 2 Divison of Infectious Diseases, Department of Medicine & Therapeutics, The Chinese University of Hong Kong, Shatin, Hong Kong, China; Fudan University, CHINA

## Abstract

**Background:**

Early initiation of antiviral therapy in elderly patients with influenza is associated with reduced risk of extra clinic visit, hospitalization and death. This study examined the cost-effectiveness of molecular POCT for detection of influenza viruses in Hong Kong elderly patients with influenza-like illness (ILI) in the outpatient clinics.

**Methods:**

A decision analytic model was used to simulate outcomes of a hypothetical cohort of elderly patients presented with ILI at outpatient clinics during peak season of influenza with two diagnostic approaches: Rapid molecular assay (POCT-PCR group) and clinical judgement with no POCT. Outcome measures included influenza-associated direct medical cost, hospitalization and mortality rates, quality-adjusted life year loss (QALY loss), and incremental cost per QALY saved (ICER).

**Results:**

In base-case analysis, POCT-PCR group was expected to reduce hospitalization (1.38% versus 2.85%) and mortality rate (0.08% versus 0.16%) and save 0.00112 QALYs at higher cost (by USD33.2 per ILI patient), comparing with clinical judgement group. The ICER of POCT-PCR was 29,582 USD/QALY saved. One-way sensitivity analyses found ICER sensitive to: Hospitalization rate without prompt antiviral therapy; odds ratio of hospitalization with prompt therapy; influenza prevalence; patient age and mortality rate of hospitalized patients. POCT-PCR was cost-effective in 60.6% and 99.4% of 10,000 Monte Carlo simulations at willingness-to-pay threshold of 1x and 3x gross domestic product per capita of Hong Kong, respectively.

**Conclusions:**

Molecular POCT for influenza detection in elderly patients with ILI at outpatient clinics during peak influenza season appeared to be cost-effective in Hong Kong.

## Introduction

The Hong Kong healthcare system is burdened by seasonal influenza every year. Influenza-associated complications lead to excess hospitalizations and deaths. In 2016, the Centre for Health Protection of Hong Kong reported in the sentinel surveillance that the annual incidence of influenza-like illness (ILI) was 30–94 per 1,000 cases at outpatient clinics and 110–300 per 1,000 cases at the accident and emergency departments [1]. The average age of patients hospitalized for influenza was over 70 years and 95% of the excess deaths occurred in elderly [2,3].

Early initiation of neuraminidase inhibitor therapy in patients with influenza is associated with reduced risk of extra clinic visit, hospitalization and death [4]. The population aged 65 years and above is growing in Hong Kong, accounted for 16.2% of 7.4 million in 2016 and projected to be 22% in 2025 [5]. Timely diagnosis of influenza and early initiation of antiviral therapy are therefore utmost critical during peak influenza season for elderly patients in Hong Kong. Real-time reverse transcriptase-polymerase chain reaction (RT-PCR) is a molecular assay providing accurate identification of influenza A and B viral RNA, yet the assay process time is 3–8 hours and it is generally not accessible in the outpatient setting. Rapid PCR molecular assays shorten the process time to 15–60 minutes, and recently became available in the outpatient setting [6,7]. The use of molecular point-of-care testing (POCT) for accurate and rapid diagnosis of influenza at the ambulatory care setting is highly desired for initiation of early and directed antiviral therapy. In this study, we examined the cost-effectiveness of molecular POCT for detection of influenza viruses in Hong Kong elderly patients with ILI in the outpatient clinics.

## Methods

### Model design

**Fig 1:**
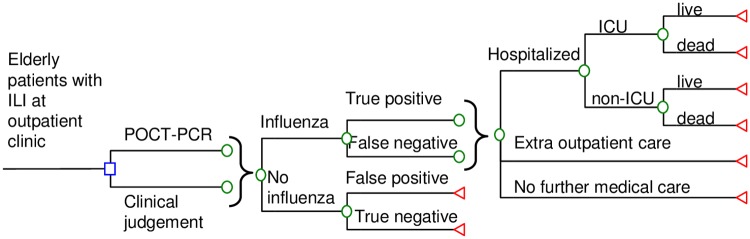
Simplified decision-analytic model.

A decision tree model (Fig 1) was designed to simulate the outcomes of a hypothetical cohort of elderly patients presented with ILI at outpatient clinics during peak season of influenza in Hong Kong. Patients who presented the ILI symptoms for over 7 days or received medical treatment in prior clinic visit(s) for the same episode of ILI were excluded. Outcomes of two diagnostic approaches were evaluated: Rapid molecular POCT (POCT-PCR group) and clinical judgement with no POCT (clinical judgement group). The model outcome measures included influenza-associated direct medical cost, hospitalization rate, mortality rate, and quality-adjusted life year loss (QALY loss).

In both arms, patients presented with ILI might or might not be infected with influenza. In the POCT-PCR arm, patients were tested using rapid PCR molecular assay. A full course of oseltamivir was prescribed to patients who were tested positive for influenza, and symptomatic treatment for those who were tested negative. In the clinical group, the initiation of oseltamivir was based solely upon clinical judgement.

After the index clinic visit, the patients infected with influenza might not seek further medical care, seek additional outpatient care, or be hospitalized. Those patients who were admitted to inpatient care might or might not receive care in the intensive care unit, and might survive or die.

### Clinical inputs

A literature search on MEDLINE over the period of 2000–2017 was performed using the keywords “influenza infection”, “neuraminidase inhibitor”, “oseltamivir”, “antiviral therapy”, “early treatment”, “outpatient visit”, “hospitalization”, “mortality” and “utility score”. The selection criteria of clinical studies of influenza infection were: (1) reports written in English; (2) etiology of ILI was identified as seasonal influenza infection, and (3) mortality rate and/or ICU admission rate were reported. All articles retrieved were screened for relevance to our model. For variable that was reported in multiple studies, the weighted average was used to estimate the base-case value and high/low values of the variable formed the range of sensitivity analysis. Subgroup data on patients aged 60 years or above from identified clinical reports were used for estimation of model inputs. When elderly subgroup data was not available, extended sensitivity analysis was performed on the non-age specific parameter by broadening the range of value.

Model inputs were shown in Table 1. Rapid molecular assays for influenza A/B reported high sensitivity (92.5%, range 87.2%-97.4%) and specificity (100%) when compared to real-time RT-PCR [7]. The accuracy of clinician judgment on influenza diagnosis in adult patients was evaluated in a prospective study at emergency department and urgent care clinic. It reported that sensitivity was 29% (95% CI 18%-43%) and specificity was 92% (95% CI 87% to 95%) [8].

**Table 1:** Model inputs.

	Base-case values	Range for sensitivity analysis	References
**Clinical inputs**			
Rapid PCR assay for influenza A/B			[7]
Sensitivity	0.925	0.872–0.974	
Specificity	1.00	-	
Clinician judgment for influenza			[8]
Sensitivity	0.29	0.18–0.43	
Specificity	0.92	0.87–0.95	
Prevalence of influenza in patients with ILI during peak season	0.192	0.109–0.257	[9]
Event rate without prompt initiation of oseltamivir			[4]
Extra clinic visit	0.478	0.465–0.491	
Hospitalization	0.018	0.0069–0.042	
With prompt initiation of oseltamivir			[4]
Odds ratio of extra outpatient visit	0.34	0.29–0.41	
Odds ratio of hospitalization	0.35	0.17–0.75	
Hospitalized for influenza			
ICU admission rate	0.105	0.031–0.164	[11]
Mortality (with ICU admission)	0.111	0.067–0.133	[2,12,13]
Mortality (without ICU admission)	0.052	0.022–0.062	[2,12,13]
**Utility inputs**			
Age	75	65–90	[5]
Utility value			
Elderly (aged 65 years and above)	0.84	-	[14]
Outpatient care	0.596	0.557–0.650	[16]
Hospitalization (non-ICU)	0.4	0.38–0.5	[16]
Hospitalization (ICU)	0.22	0.176–0.264	[15]
Duration of illness (days)			
Length of illness in outpatient care	7	3–11	[17]
Accelerated factor with early oseltamivir	0.56	0.42–0.76	[10]
Length of hospitalization in ICU	7.9	1.9–13.9	[2]
Length of hospitalization in non-ICU units	5.5	2.8–8.2	[2]
**Cost inputs (USD)**			
Cost per rapid PCR test	48	38–58	[19]
Oseltamivir (full course)	24	19–29	Local
Symptomatic treatment	5	3.8–6.4	Local
Cost per outpatient clinic visit	50	40–60	[20]
Cost per hospitalization for elderly patients with influenza			
without ICU care	5,773	3,756–7,790	[2]
with ICU care	36,588	15,106–58,070	[2]

The prevalence of influenza among patients presented with ILI during peak season was estimated from the weekly statistics on detection of influenza viruses from respiratory specimens reported by the Centre for Health Protection in 2016 [9]. The weekly prevalence in January to May 2016 were higher than 10% and the weighted average was estimated to be 19.2% (range 10.9%-25.7%). The outcomes (measured as extra clinic visit and hospitalization) of promptly initiation of oseltamivir therapy within one week were reported in an observational study conducted in an Asian population cohort. The weighted average rates of extra clinic visit and hospitalization in elderly patients (age groups 60 years to 80+ years) without prompt initiation of oseltamivir was estimated to be 47.8% (range 46.5%-49.1%) and 1.8% (range 0.69%-4.2%), respectively. Prompt oseltamivir therapy was reported to reduce the odds for extra outpatient visit (OR 0.34; 95% CI 0.29–0.41) and hospitalization (OR 0.35; 95% CI 0.17–0.75) in the elderly age groups [4]. Early oseltamivir treatment was reported to reduce the duration of clinical illness (adjusted accelerated factor 0.56; 95% CI 0.42–0.76) in a prospective clinical trial conducted in Hong Kong [10]. The event rates in elderly patients hospitalized for seasonal influenza were reported in observational studies in Hong Kong. The ICU admission rate (10.5%) [11] and mortality rate (11.1% in ICU and 5.2% in non-ICU) in elderly patients hospitalized for seasonal influenza were retrieved from outcome analyses of severe influenza infection [2,12,13].

### Utility inputs

The QALY loss for influenza infection was calculated using the disutility (difference between utility values of elderly and the event), and the duration of time-spent in each of following events: (1) outpatient care; (2) hospitalization without ICU admission; (3) ICU care; and (4) influenza-related death. The utility value of each health status was estimated from findings of health-related quality of life studies [14–16]. The duration of illness (as time-spent) for outpatient care [17] and length of hospital stay with and without ICU admission (reported by an outcome study of hospitalized elderly patients for influenza) [2] were used to estimate the QALY loss. The QALY loss as a result of death was calculated using projected age-specific life expectancy [18] reported in Hong Kong life-table and age-specific utility value [11], discounted by an annual rate of 3%. The weighted average age of Hong Kong elderly was 75 years (range 65–90 years), estimated from the projected 2017 mid-year population by age group [5], and it was used as the base-case value of age in the present hypothetical cohort.

### Cost inputs

The economic analysis of direct medical cost was conducted from the perspective of healthcare provider in Hong Kong. Cost items included rapid molecular test, oseltamivir (75mg orally twice daily for 5 days), symptomatic treatment, outpatient clinic visit, hospitalization with and without ICU care. The cost per rapid molecular test (USD48; USD1 = HKD7.8) for influenza was adopted from literature [19]. The costs of oseltamivir and symptomatic treatment (including analgesic, antipyretics, and common cold products) were estimated from drug acquisition costs in Hong Kong. The cost per general outpatient clinic visit was estimated from the 2017 charge per general outpatient clinic of Hospital Authority [20]. Hospital Authority is the largest, non-profit-making public health organization in Hong Kong providing primary to tertiary care. The charges listed represent only the cost components (including labor costs) with no addition of profits, and it was therefore adopted for estimation of cost of health service from provider’s perspective.

### Cost-effectiveness analysis

Expected value of each outcome measure (influenza-associated direct medical cost, hospitalization rate, mortality rate, and QALY loss) was simulated with the base-case model input values. Comparing with clinical group, if the POCT-PCR group saved QALYs at higher cost, incremental cost of per QALY saved (ICER) would be calculated using the following equation: (Cost_POCT-PCR_ − Cost_clinical judgement_)/(QALY loss_clinical judgement_ − QALY loss_POCT-PCR_). As recommended by the World Health Organization (WHO), ICER less than 1x gross domestic product (GDP) per capita was considered as highly cost-effective and ICER less than 3x GDP per capita was cost-effective. The GDP per capita of Hong Kong was USD43,497 in 2016 [21] and it was therefore used as the threshold of willingness-to-pay (WTP) per QALY [22]. A strategy with ICER less than 43,497 USD/QALY was considered as highly cost-effective.

Sensitivity analysis was performed by TreeAge Pro 2009 (TreeAge Software, Inc., Williamstown, MA, USA) and Microsoft Excel 2010 (Microsoft Corporation, Redmond, WA, USA) to examine the robustness of the model results. The range for sensitivity analysis was the 95% CI range, high/low values of the variable or +/- 20% of the base-case value. One-way sensitivity analysis on all model inputs was performed to identify influential factors with threshold value.

Some model inputs were not age-specific to elderly patients or Hong Kong practice, and extended sensitivity analysis was conducted on such parameters with broadened ranges. The sensitivity and specificity of clinical judgement were retrieved from a study conducted in the US [8]. Variation in clinical and laboratory training at different countries imposed uncertainty on the transferability of these variables to the present Hong Kong model. Extended one-way sensitivity analysis was performed on the sensitivity and specificity of clinical judgement and POCT-PCR over 0%-100%. The effectiveness of prompt oseltamivir in Hong Kong elderly patients were obtained from one Asian population cohort study with elderly subgroup data [4] and one Hong Kong cohort study (age-specific data not available) [10]. Systematic reviews only found modest reduction of length of symptoms by antivirals [23,24]. To examine the impact of antiviral effectiveness on the robustness of present analysis, the odds ratios of events (extra outpatient visit and hospitalization) [4] and accelerated factor on length of illness with prompt initiation of oseltamivir [10] were therefore also examined in extended one-way sensitivity analysis. The value of 1 (same odds of event and length of illness as no prompt treatment) was used as the upper limit of variation. The Hong Kong surveillance data of influenza viruses did not differentiate the prevalence of influenza among patients presented with ILI by age groups [9]. Extended sensitivity analysis was performed on this parameter over 0%-100%.

To evaluate the impact of uncertainty in all variables simultaneously, a probabilistic sensitivity analysis was performed using Monte Carlo simulation. The direct cost and QALY loss of POCT-PCR and clinical judgement arms were recalculated 10,000 times by randomly drawing each of the model input from a triangular probability distribution within the range for sensitivity analysis. The probability of each study arm to be the preferred option was determined over a wide range of 1-3x GDP per capita as threshold of WTP.

## Results

### Base-case analysis

The base-case analysis results were shown in Table 2. Comparing with the clinical judgement group, the POCT-PCR group was expected to reduce hospitalization and mortality rate and save QALY at higher cost. The ICER of POCT-PCR versus clinical judgment was 29,582 USD/QALY saved, lower than the WTP threshold (43,497 USD/QALY).

**Table 2:** Base-case analysis of influenza-related direct medical cost, infection rate, mortality rate and QALY loss.

Strategy	Clinical judgement	POCT-PCR
Cost per elderly (USD)	83.4	116.6
Hospitalization rate per 1000 elderly	2.80	1.38
Mortality rate per 1000 elderly	0.16	0.08
QALY loss per elderly	0.00251	0.00139
Increment cost of rapid PCR versus clinical judgment		
per elderly	-	33.2
at elderly population^a^	-	40,603,600
QALY saved by rapid PCR versus clinical judgment		
per elderly	-	0.00112
at elderly population^a^	-	1,370
ICER (USD/QALY saved)^b^	-	29,582

ICER = incremental cost-effectiveness ratio; QALY = quality-adjusted life year

^a^Hong Kong elderly (65–90 years) population projected to be 1,223,000 in mid-2017 [5].

^b^POCT-PCR group was highly cost-effective when ICER was less than 43,497 USD/QALY (gross domestic product per capita in Hong Kong) (USD1 = HKD7.8)

### Sensitivity analysis

One-way sensitivity analysis found that the POCT-PCR group remained QALY-saving at higher cost throughout variation of all model inputs over the range for sensitivity analysis. To examine the impact of lowering cost per rapid-PCR test on the total cost, the range for sensitivity analysis (USD38-58) was extended to USD10-58. The extended one-way sensitivity analysis found a threshold value that the total cost in POCT-PCR was lower than clinical judgement group when the cost per rapid molecular assay was <USD14.8.

**Fig 2:**
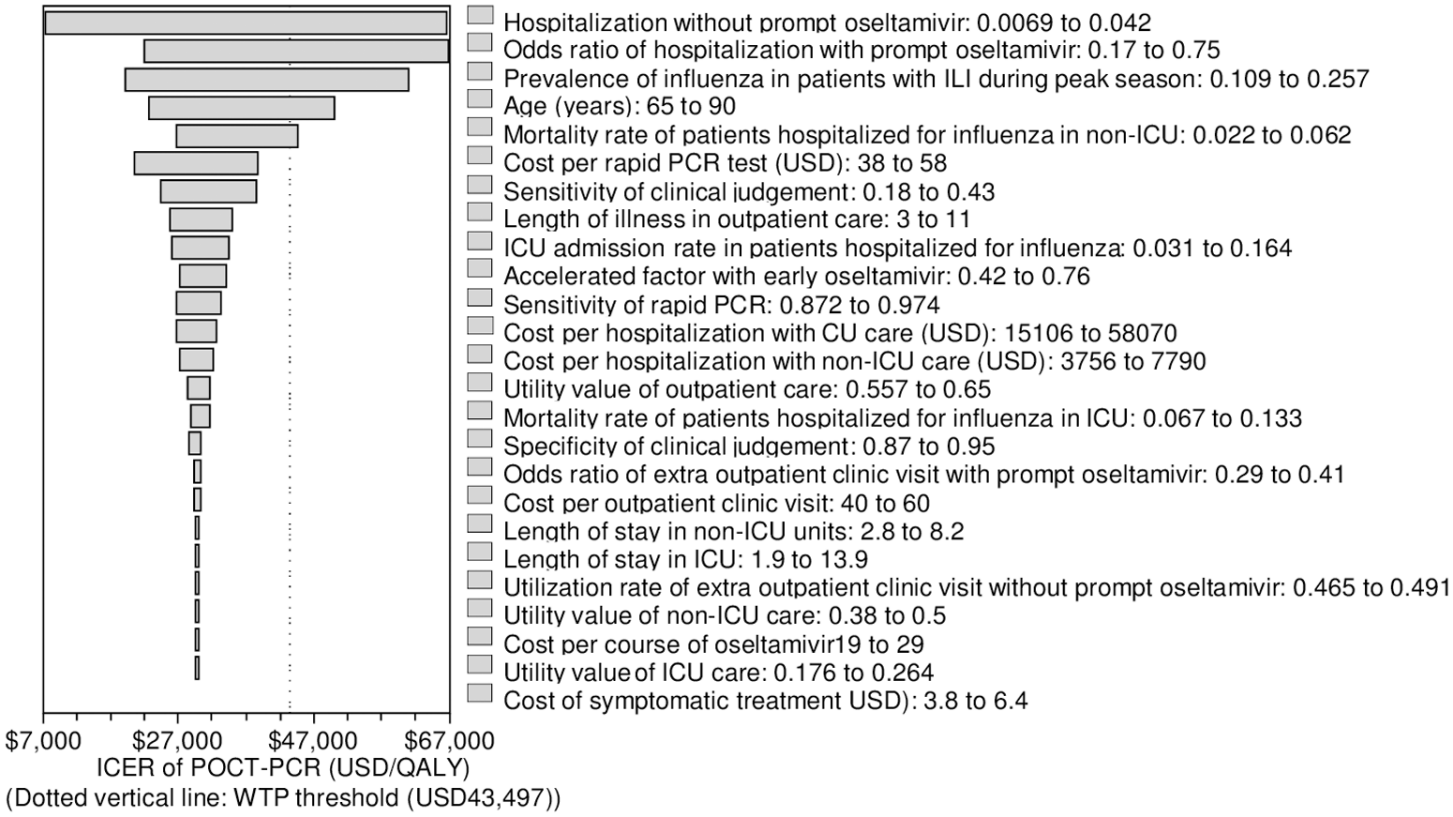
One-way sensitivity analysis (tornado diagram) of all parameters on ICER of POCT-PCR.

The impact of one-way variation of all parameters on ICER of POCT-PCR was shown in a tornado diagram (Fig 2). The acceptance of POCT-PCR to be highly cost-effective (ICER<1x GDP per capita) was sensitive to five influential factors: Hospitalization rate in elderly without prompt oseltamivir therapy; odds ratio of hospitalization in elderly with prompt oseltamivir therapy; prevalence of influenza in elderly patients with ILI; age of patients and mortality rate of elderly patients admitted to non-ICU ward for influenza. In the extended one-way sensitivity analysis, specificity of clinical judgement and POCT-PCR sensitivity were found to be influential. The threshold value of each influential factor was shown in Table 3. The ICER of POCT-PCR would remain below 1x GDP per capita if the value of each influential factor was below (or above) the threshold values as indicated in Table 3.

**Table 3:** Threshold values of influential factors on ICER of POCT-PCR identified in one-way and extended one-way sensitivity analysis.

Variables	Base-case value	Range for sensitivity analysis	Threshold value for ICER of POCR-PCR <1x GDP per capita
One-way sensitivity analysis			
Hospitalization rate without prompt initiation of oseltamivir	0.018	0.0069–0.042	>0.012
Prevalence of influenza in patients with ILI during peak season	0.192	0.109–0.257	>0.143
Odds ratio of hospitalization with prompt initiation of oseltamivir	0.35	0.17–0.75	<0.559
Age	75	65–90	<87
Mortality (without ICU admission)	0.052	0.022–0.062	>0.023
Extended one-way sensitivity analysis	Base-case value	Extended range for sensitivity analysis	Threshold value for ICER of POCR-PCR <1x GDP per capita
Specificity of clinician judgment for influenza	0.29	0–1	<0.493
Sensitivity of POCT-PCR for influenza	0.925	0.872–0.974	>0.754

**Fig 3:**
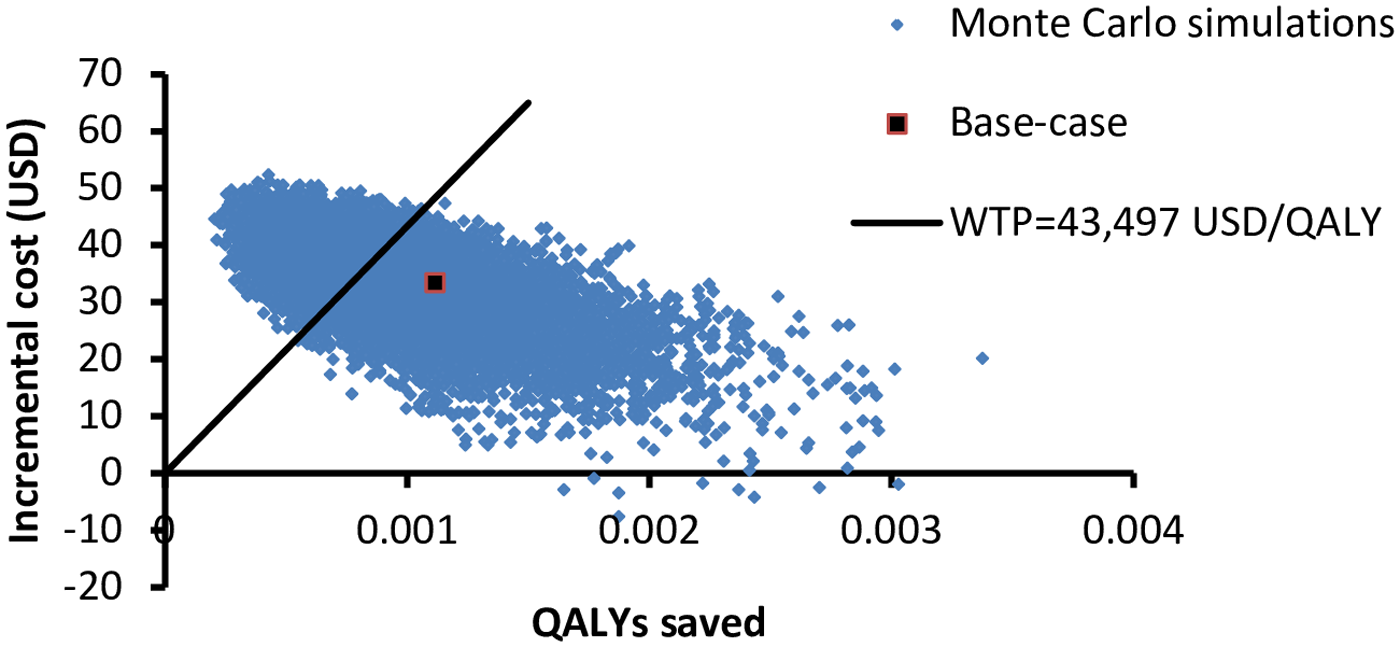
Scatter plot of incremental cost versus QALYs saved by POCT-PCR group versus clinical judgment group.

The probabilistic sensitivity analysis was performed by 10,000 Monte Carlo simulations. A scattered plot (Fig 3) showed the incremental cost versus QALYs saved by the POCT-PCR group. Comparing to the clinical judgement group, the POCT-PCR group saved QALYs in 100% of the time and the mean QALYs saved was 0.000979 QALYs (95%CI 0.000971–0.000987; p <0.001). The POCT-PCR program was more costly in 99.91% of the simulations and the mean additional cost of POCT-PCR was USD32.7 (95%CI 32.5–32.8; p<0.001). The ICERs were above the WTP threshold (1x GDP per capita) in 39.5% of simulations.

**Fig 4:**
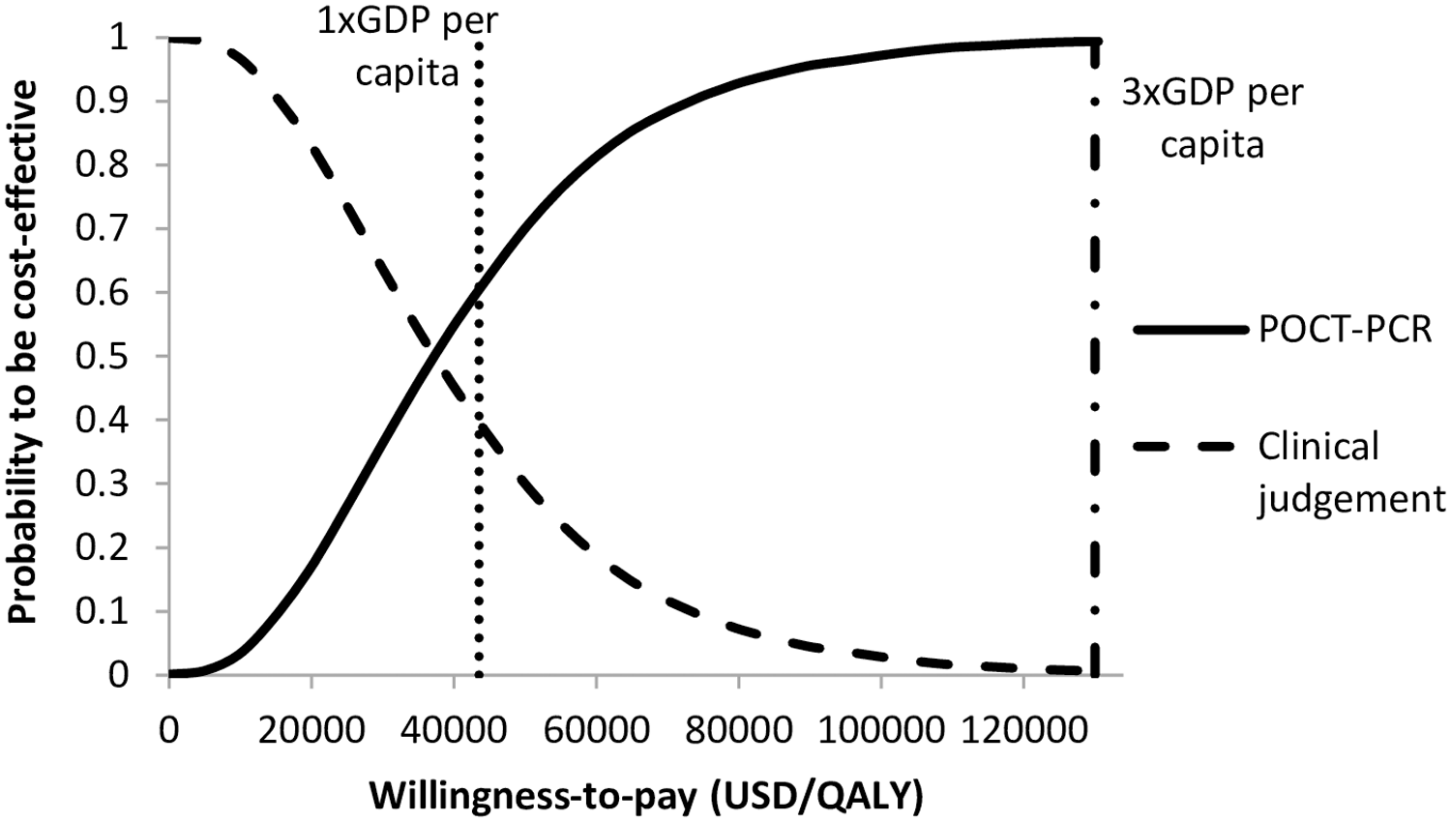
Probabilities of POCT-PCR and clinical judgment to be accepted as cost-effective against willingness-to-pay.

The probabilities of POCT-PCR group to be the preferred option were examined in the acceptability curve over a wide WTP range (0 to 3x GDP per capita) (Fig 4). Using 1x GPD per capita (USD43,497) as WTP threshold, the POCT-PCR was the preferred option in 60.6% of 10,000 Monte Carlo simulations. If the WTP was 3x GDP per capital (USD130,491), the probability of POCT-PCR to be accepted as cost-effective increased to 99.4%.

## Discussion

Our study investigated the cost-effectiveness of applying rapid molecular assay as POCT for detecting influenza at the ambulatory care setting. The base-case findings showed that POCT-PCR saved QALYs by reducing the rates of subsequent hospitalization for influenza and mortality. The POCT-PCR incurred higher total direct cost when compared to the clinical judgement group by USD33.2 per patients tested. The expected ICER of POCT-PCR was USD29,582, lower than 1x GDP per capita of Hong Kong (USD43,497). The base-case ICER was therefore highly cost-effective from the perspective of healthcare provider in Hong Kong.

The one-way sensitivity analysis found the base-case results on QALYs saved and incremental cost incurred by POCT-PCR to be robust. POCT-PCR remained to save QALYs at higher cost than the clinical judgement group throughout variation of all model parameters. The acceptance of POCT-PCR to be highly cost-effective (ICER <1x GDP per capita) was sensitivity to variation of five influential factors. Four of the five factors were model inputs on outcomes of hospitalization for influenza: Hospitalization rate without prompt initiation of oseltamivir, odds ratio of hospitalization with prompt oseltamivir, mortality rate in hospitalized patients, and age. These four factors represented the effectiveness of prompt initiation oseltamivir (as a result of accurate diagnosis of influenza in the early state of infection) in prevention of hospitalization. The effectiveness in reduction of hospitalization for influenza was highly influential on the POCT-PCR cost-effectiveness. The costs per hospitalized case of seasonal influenza in elderly with and without ICU were reported to be substantial in Hong Kong [2], and the mortality rate of elderly patients hospitalized for seasonal influenza was high [2,12,13]. The threshold of influenza prevalence was 14.3% or above for the POCT-PCR group to be cost-effective for obvious reason that relatively high level of influenza circulation was required for cost-effective usage of a costly test (USD48 per test). Extended one-way sensitivity analysis further identified the threshold values of specificity of clinical judgment and POCT-PCR sensitivity required for POCT-PCR to be preferred over clinical judgment. Probabilistic sensitivity analysis demonstrated that the POCT-PCR group was highly cost-effective (ICER< 1x GDP per capita) in 60.6% and cost-effective (ICER< 3x GDP per capita) in 99.4% of 10,000 Monte Carlo simulations.

A health economic study compared the direct medical costs of applying in-house rapid PCR test for detecting influenza in patients with ILI at the emergency department and in hospitalized patients [19]. The findings showed that rapid PCR assay was cost-saving by reducing the resources (assay reagents and technical staff, disposable, healthcare personnel) used in examination room and isolation room of emergency department. Both the reported economic findings and our results supported rapid PCR assay to be a potentially cost-effective POCT for early treatment of influenza infection. Our findings further evaluated the potential QALYs saved by reducing duration of illness, hospitalization, and mortality, as a result of early detection and treatment of influenza in elderly patients at the outpatient clinics.

Influenza vaccine is the recommended measure for prevention of influenza, yet the vaccination coverage in Hong Kong elderly (39.1%) has been lower than optimal [25]. Near-patient assays allowed early detection and therefore timely treatment for patients with influenza. Early treatment of influenza within 48 hours of symptom onset was associated with lower risk for developing complications such as pneumonia and respiratory failure [26,27]. Prompt initiation of antiviral therapy (within one-week) was also associated with reduced usage of additional outpatient patient care, hospitalization and death [4,12]. POCT-PCR for influenza can assist and enable more effective use of antiviral treatment by early detection of disease [28].

The present study was limited by intrinsic limitations of decision-analytic modeling that the model results were subject to uncertainties of model inputs. Sensitivity analyses were therefore conducted by one-way variation of each input and simultaneous variation of all parameters to examine the robustness of base-case results. The real-life events of ILI and influenza infection were simplified in the model. Different rapid PCR assays are categorized to various levels of technical complexity. The accuracy of technical skills in conducting the POCT-PCR assay might be associated with the level of product complexity, affecting the transferability of POCT-PCR sensitivity and specificity from clinical trial to daily practice. Variation of medical training programs in different countries also limited the transferability of overseas sensitivity and specificity of clinical judgment to Hong Kong. The cost and QALY loss in clinical judgment arm might be overestimated in some false-negative cases which are sub-clinical types of influenza with low hospitalization rate. The transmission of infection from influenza patients to uninfected subjects and misused of antibiotics for influenza patients were not included. The economic benefits and QALYs saved by early detection and treatment of influenza using POCT-PCR might therefore be underestimated.

In conclusion, using rapid molecular assay as POCT for detection of influenza in elderly patients with ILI (for less than 7 days) at outpatient clinics during period with high circulation of influenza viruses appeared to be a cost-effective option to reduce hospitalization and mortality rate, and save QALYs from the perspective of healthcare provider in Hong Kong. The cost-effectiveness of POCT-PCR was subject to the threshold of WTP, age of elderly, prevalence of influenza, baseline hospitalization rate without prompt antiviral therapy, effectiveness of prompt initiation of antiviral therapy to reduce hospitalization, mortality rate of hospitalized elderly for influenza, and accuracy of clinical judgement and PCR for influenza.
